# Non-ribosomal peptides produced by *Planktothrix agardhii* from Siemianówka Dam Reservoir SDR (northeast Poland)

**DOI:** 10.1007/s00203-014-1008-9

**Published:** 2014-06-28

**Authors:** Magdalena Grabowska, Justyna Kobos, Anna Toruńska-Sitarz, Hanna Mazur-Marzec

**Affiliations:** 1 Department of Hydrobiology, University of Białystok, Świerkowa 20B, 15-950 Białystok, Poland; 2 Institute of Oceanography, University of Gdańsk, Al. Marszałka Piłsudskiego 46, 81-378 Gdynia, Poland

**Keywords:** *Planktothrix agardhii*, Microcystins, Non-ribosomal peptides, Reservoir

## Abstract

*Planktothtrix agardhii* (Oscillatoriales) is a filamentous cyanobacterium, which frequently forms blooms in shallow, polymictic and eutrophicated waters. This species is also a rich source of unique linear and cyclic peptides. In the current study, the profile of the peptides in samples from the *P. agardhii*-dominated Siemianówka Dam Reservoir (SDR) (northeast Poland) was analyzed for four subsequent years (2009–2012). The LC–MS/MS analyses revealed the presence of 33 peptides. Twelve of the most abundant ones, including five microcystins, five anabaenopeptins, one aeruginosin and one planktocyclin, were present in all field samples collected during the study. The detection of different peptides in two *P. agardhii* isolates indicated that the SDR population was composed of several chemotypes, characterized by different peptide patterns. The total concentration of microcystins (MCs) positively correlated with the biomass of *P. agardhii*. Between subsequent years, the changes in the ratio of the total MCs concentration to the biomass of *P. agardhii* were noticed, but they were less than threefold. This is the first study on the production of different classes of non-ribosomal peptides by freshwater cyanobacteria in Poland.

**Electronic supplementary material:**

The online version of this article (doi:10.1007/s00203-014-1008-9) contains supplementary material, which is available to authorized users.

## Introduction

Cyanobacteria, as other groups of bacteria, produce a wide range of non-ribosomal peptides (Welker and von Döhren 2006; Sivonen and Börner 2008). These metabolites were identified in bloom samples from fresh, brackish and marine waters and in many isolated strains, mainly from *Microcystis*, *Planktothrix* and *Dolichospermum* (*Anabaena)* genera. The occurrence of the cyclic heptapeptides called microcystins was reported most frequently. These compounds belong to protein phosphatase inhibitors and show a strong hepatotoxic activity. Due to numerous incidents of human and animal poisoning, the presence of microcystins in drinking water resources is of serious public health concern (Sivonen and Jones 1999). Other classes of non-ribosomal peptides that are commonly found in cyanobacteria include anabaenopeptins, aeruginosins, cyanopeptolines and microginins (Welker and von Döhren 2006). Within each class of the compounds, several structural variants were identified. The production of specific peptide classes and their variants is genetically determined. The profile of the compounds was generally thought to be a stable and unique feature of an individual strain, and it was used to distinguish metabolically diverse subpopulations (chemotypes) occurring in the same reservoir (Rohrlack et al. 2008).

Studies conducted in different freshwater bodies in Europe, including lakes Maxsee in Germany (Welker et al. 2004a), Steinfjorden in Norway (Rohrlack et al. 2008) and Zürich in Austria (Sogge et al. 2013) showed that the number of coexisting *Planktothrix* chemotypes can range from 4 to 18, depending on the lake (Yépremian et al. 2007; Bauman and Jüttner 2008; Rohrlack et al. 2008). In some of the lakes, the composition of *Planktothrix* chemotypes was relatively stable, even over a long time period (Rohrlack et al. 2009). Over a 33-year persistence of four *Planktothrix* chemotypes was documented in Lake Steinsfjorden by Rohrlack et al. (2008, 2009). Also, Bauman and Jüttner (2008), during 4-year studies, revealed the presence of the same peptide variants in *Planktothrix* samples from Lake Hallwilersee. According to the authors, these results indicated the stability of the chemotype composition in the lake.

In our work, the diversity of non-ribosomal peptides produced by cyanobacteria from Polish freshwater body was studied for the first time. For the purpose of the study, the Siemianówka Dam Reservoir (SDR), northeast Poland, was selected. This artificial reservoir was constructed in the upper part of the Narew River in 1990. In the shallow polymictic and highly eutrophicated SDR, the dominance of cyanobacteria in phytoplankton community in summer and autumn has always been observed (Grabowska 2005). During the first 12 years (1992–2003), the representatives of three orders of cyanobacteria co-occurred: Nostocales (*Aphanizomenon, Dolichospermum (Anabaena)),* Chroococcales (*Microcystis, Woronichinia*) and Oscillatoriales (*Limnothrix, Planktothrix, Pseudanabaena*). In 2006, for the first time, the intensive growth of *Planktothrix agardhii* in phytoplankton community was recorded. In October 2006, the biomass of the species increased 8 times compared to 2005 and exceeded 50 mg/l (Grabowska and Pawlik-Skowrońska 2008). Since 2006, a clear dominance of *P. agardhii* has been established. From early spring to late autumn, and sometimes in winter, the species usually constituted over 90 % of the phytoplankton biomass (Grabowska and Mazur-Marzec 2011).

In the present study, the structure and profile of peptides produced by the cyanobacteria from the SDR, dominated by *P. agardhii*, were characterized. In addition, the changes in the peptide pattern in the SDR in four subsequent years (2009–2012) and throughout the whole growth season were examined. The oligopeptide profile in the reservoir was compared with the profiles of the metabolites characteristic for *P. agardhii* populations from other European water bodies.

## Materials and methods

### Sampling and analysis of phytoplankton

The studies into the production of cyanopeptides were conducted in the SDR located in the northeast part of Poland (52°55′N, 23°50′E). The surface water samples (0.5 m) were collected with the Limnos sampler. In 2010 and 2011, the studies were carried out from May to October. In 2009 and 2012, the number of sampling days was limited to 1 and 3, respectively (Table 1). Water samples (0.5–1.0 l) for the analyses of peptides were passed through Whatman GF/C glass microfiber filters. Then, the filters were stored frozen. Material for microscopic analysis was preserved by the addition of 0.3 ml acidified Lugol’s solution to 100-ml sample. A light microscope (Olympus BX50) was used for qualitative analyses. Phytoplankton abundance was determined according to the Utermöhl method (Utermöhl 1958) using an inverted microscope (Olympus CX 41). The biovolume was calculated by multiplying the number of individuals (cell, coenobium, colony or 100 µm filament) of the particular taxa by their volume measured according to Hillebrand et al. (1999). Assuming that the density of organisms is equal to water (1.0 g/ml), the biomass (wet weight) was estimated as: 1 mm^3^/l = 1 mg/l (Rott 1981).

**Table 1:** Biomass of *Planktothrix agardhii* and microcystins (MCs) concentration in the SDR in years 2009–2012

Date	Tw (°C)	*Planktothrix agardhii*		MCs Concentration (µg/l)	MC/Biomass (×10^−3^)
		Biomass (µg × 10^3^/l)	% in cyano-biomass		
05/10/09	11.3	34.2	98.0	8.3	0.24
31/05/10	17.1	14.7	97.5	4.7	0.32
30/06/10	23.0	15.5	85.2	5.5	0.35
12/07/10	26.4	27.0	90.6	7.2	0.27
09/08/10	24.4	34.5	87.4	9.6	0.28
23/08/10	21.8	38.4	96.0	12.2	0.32
13/09/10	15.7	69.7	95.3	23.1	0.33
27/09/10	14.7	28.3	97.3	8.3	0.29
04/10/10	12.2	36.3	97.3	8.9	0.24
19/10/10	7.1	26.5	99.9	6.3	0.24
24/05/11	18.7	9.4	86.7	6.5	0.69
18/07/11	25.4	15.8	88.7	9.5	0.60
18/08/11	21.6	15.5	96.9	9.3	0.60
21/09/11	18.4	44.8	98.8	22.4	0.50
13/10/11	11.6	8.4	99.3	11.4	1.36
17/07/12	21.8	18.5	78.0	4.5	0.34
21/08/12	19.0	30.4	91.8	17.3	0.57
16/10/12	9.8	28.1	96.9	10.5	0.37

### Isolation and taxonomic identification of the isolates

Two strains of *P. agardhii* were isolated from the bloom sample collected from the SDR on October 16, 2012. The isolates were identified using microscopic analysis (Komárek and Anagnostidis 2005) and molecular methods. Single trichomes of *P. agardhii* were picked up and purified by multiple transfers to agar (1.0 % bacterial agar) and liquid Z8 medium (Kotai 1972). The isolates were cultivated at 22 °C, and continuous light of 5 µE m^2^/s provided by standard cool white fluorescent lamps. The strains, CCNP1325 and CCNP1326, were deposited in the Culture Collection of Northern Poland at the Institute of Oceanography, University of Gdańsk. Genomic DNA was extracted with Genomic Mini Kit (A&A Biotechnology), according to the manufacturer’s instructions. 16S rRNA gene cyanobacteria-specific primers were used: CYA359F (Nübel et al. 1997) and 23S30R (Lepére et al. 2000). The PCR was performed according to Koskenniemi et al. (2007) with minor changes (annealing temperature changed to 57 °C). The amplified PCR products were purified using Clean-up Kit (A&A Biotechnology). Nucleotide sequences have been deposited in the GenBank database under the accession numbers KF976399 for CCNP1325 and KF976400 for CCNP1326.

### Extraction and analysis of cyanobacterial peptides

Cyanobacterial material collected on filters or the scums (0.5 ml) were extracted with 1 ml of 90 % methanol in water by 10-min bath sonication (Sonorex, Bandeline, Berlin, Germany) followed by 1-min probe sonication with an ultrasonic disrupter HD 2070 Sonopuls (Bandeline, Berlin, Germany). After centrifugation at 10,000 *g* for 15 min, the supernatants were transferred to chromatographic vials.

The analyses of cyanobacterial extracts were performed with Agilent 1200 (Agilent Technologies, Waldboronn, Germany) coupled online to a hybrid triple quadrupole/linear ion trap mass spectrometer (QTRAP5500, Applied Biosystems, Sciex; Concorde, ON, Canada). As a mobile phase, a mixture of A (5 % acetonitrile in water containing 0.1 % formic acid) and B (100 % acetonitrile containing 0.1 % formic acid) was used. Separation was performed on a Zorbax Eclipse XDB-C18 column (4.6 × 150 mm; 5 µm) (Agilent Technologies, Santa Clara, California, USA). Phase B was linearly increased from 15 to 75 % in 5 min and then to 90 % in the next 5 min. This composition of the mobile phase was held for 5 min and brought back to 15 % B in 1 min. The column oven temperature was 35 °C, the flow rate was 0.6 ml/min and the injection volume was 5 μl.

The structures of cyanobacterial peptides were characterized using the QTRAP LC–MS/MS system equipped with a turbo ion source (550 °C; 5.5 kV). The experiments were run in a positive mode using the information-dependent acquisition method (IDA). In addition, the enhanced ion product spectra (EIP) were acquired from 50 to 1,200 Da with a collision energy (CE) of 60 V and collision energy spread (CES) of 20 V. Declustering potential (DP) was set at 80. Data acquisition and processing were accomplished using Analyst QS^®^ 1.5.1 software.

For the quantitative analysis of microcystins, the HPLC system (Agilent 1200, Agilent Technologies, Waldboronn, Germany) equipped with a photodiode array detector (PDA) was used; the absorbance at 238 was monitored. The separation was performed on a Luna RP-18 column (3.0 mm × 150 mm; 3 µm) kept at temperature of 30 °C. Gradient elution with the mobile phase A (5 % acetonitrile in MilliQ water with 0.05 % trifluoroacetic acid TFA) and B (100 % acetonitrile with 0.05 % TFA) was used. The mobile phase was delivered at a flow rate of 0.5 ml/min. Phase B was linearly increased from 30 to 70 % in 7 min and then to 100 % in 3 min. The column was washed with 100 % phase B for 10 min, and then, the mobile phase composition was brought back to the initial conditions (70 % B) in 1 min. The total concentration of microcystins in the analyzed samples was calculated as MC-LR equivalents using a calibration curve prepared with a standard of MC-LR (Alexis Biochemicals, Lausen, Switzerland).

### Statistical analysis

Statistical analyses as Spearman correlations and one-way ANOVA test were run with STATGRAPHICS 1.4 PL software.

## Results and discussion

### Cyanobacteria in SDR

The analyses of phytoplankton samples collected from the SDR in years 2009–2012 confirmed the established dominance of *P. agardhii* in the reservoir (Grabowska and Mazur-Marzec 2011). From May to October, the biomass of *P. agardhii* ranged from 9.4 mg/l (24 May 2011) to 69.7 mg/l (13 September 2010) and it constituted 78.0 % (July 2012) to 99.9 % (October 2010) of the total cyanobacterial biomass (Table 1). Among other cyanobacteria, mainly the representatives of Oscillatoriales (*Limnothrix redekei*, *Pseudanabaena limnetica*, *Planktolyngbya* spp.) and Nostocales (*Aphanizomenon gracile, Aph. flos*-*aquae, Aph. issatschenkoi, Dolichospermum circinalis, D. planctonicum, D. flos*-*aquae*) were present in the SDR.

Cyanobacteria belonging to *Planktothrix* genus are quite common in eutrophicated waters of many European countries. The blooms of *Planktothrix* were recorded in Germany (Welker et al. 2004a), Austria, the Netherlands and Denmark (Kurmayer et al. 2011), Belgium, Luxembourg (Willame et al. 2006) and France (Yépremian et al. 2007). In Poland, *Planktothrix* was present in 38 % of the 238 examined lakes, and in 17 % of the lakes, it dominated or codominated (Kobos et al. 2013). Due to well-developed adaptive strategies, this bloom-forming species can persist in a wide range of temperature conditions. It can also grow in turbid waters, at lower light intensity.

In this work, two strains of *P. agardhii* were isolated from SDR in October 2012: CCNP1325 and CCNP1326. According to BLAST search, the 16S rRNA sequences (636 bp) were highly similar (100 %) to other sequences from *P. agardhii* and *P. rubescens* isolates from European and Asian water bodies.

### Non-ribosomal peptides produced by cyanobacteria in SDR

**Fig. 1:**
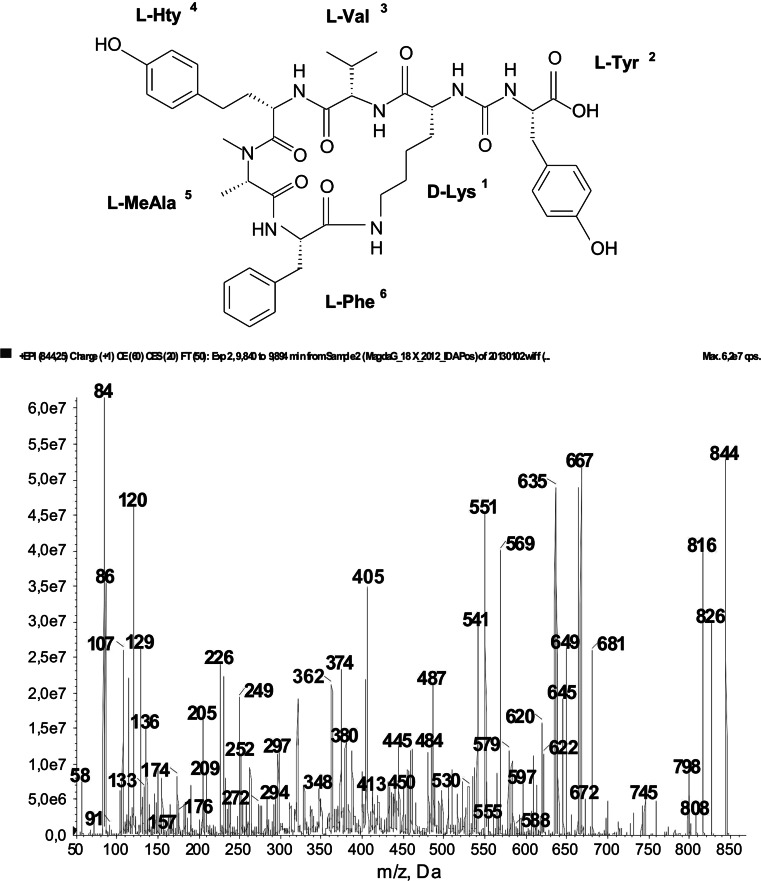
Chemical structure and enhanced ion product mass spectrum of anabaenopeptin A ([Phe-MeAla-HTyr-Val-Lys]CO-Tyr), with [M + H] ion at *m/z* 844, produced by *Planktothrix agardhii* from the SDR. The mass signals were assigned to the following fragments: 826 [M + H − H_2_O], 816 [M + H − CO], 798 [M + H − H_2_O − CO], 745 [M + H − Val], 681 [M + H − Tyr], 667 [M + H − Hty], 635 cyclo[Lys-Val-Hty-MeAla-Phe − H], 569 [M + H − (Hty − Val)], 551 [M + H − (Hty − Val) − H_2_O], 405 [Htyr-Val-Lys + 2H], 362 [Ile-Hty-MeAla + H], 320 [(Lys-CO-Phe) + H], 136 Tyr-immonium ion, 107 [CH_2_PhOH], 84 Lys-immonium ion, 58 MeAla-immonium ion

**Fig. 2:**
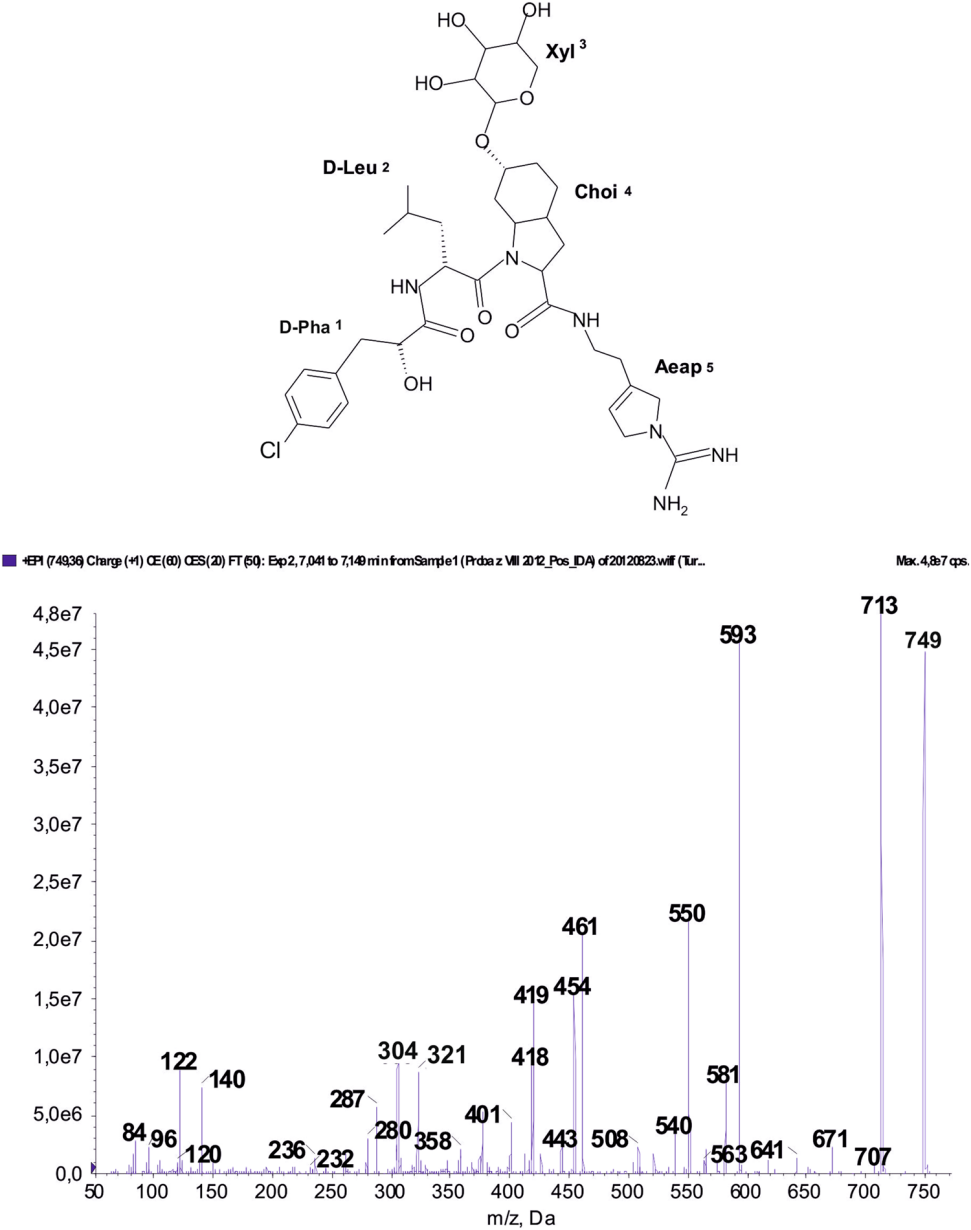
Chemical structure and enhanced ion product mass spectrum of aeruginosin (Cl-Pla-Leu-(Xyl)Choi-Aeap), with [M + H] ion at 749, produced by *Planktothrix agardhii* from the SDR. The mass signals were assigned to the following fragments: 713 [M + H − Cl], 707 [M + H − CH_2_N_2_], 671 [M + H − CH_2_N_2_ − Cl], 593 [M + H − Cl − Pla fragment], 581 [M + H − Cl − Xyl], 461 [M + H − Cl − Pla fragment – Xyl], 454 [(Xyl)Choi-Aeap + 2H], 322 [Choi-Aeap + 2H], 305 [Choi-Aeap + H − NH_2_], 280 [Choi-Aeap + 2H − CH_2_N_2_], 140 Choi-immonium ion, 122 Choi-immonium − H_2_O

*Planktothrix* is considered to be an effective producer of microcystins and many other oligopeptides (Kurmayer et al. 2005; Welker et al. 2004a; Rohrlack et al. 2009). In the cyanobacterial material collected from the SDR, 33 oligopeptides including microcystins, anabaenopeptins (Fig. 1), aeruginosins (Fig. 2), and also single representatives of aeruginosamides (Fig. 1S), cyanopeptolines and planktocyclins (Fig. 2S) were detected (Table 2).

**Table 2:** Peptide profile of *Planktothrix agardhii* samples from the SDR and of two strains isolated from the reservoir: *CCNP1325* and *CCNP1326*

No	[M + H]^+^ *m/z*	Structure of oligopeptide	2009	2010									2011				2012			CCNP1325	CCNP1326
			05/10	31/05	30/06	12/07	09/08	23/08	13/09	27/09	04/10	19/10	24/05	18/07	18/08	21/09	17/07	21/08	16/10		
1	1126.6	PP GA-HTyr-Gln-Thr-Leu-Aph-Thr-diMetyr-Ile																	x		
2	1045.5	MC [Asp^3^, Mdha^7^]MC-HtyR	x	x	x	x	x	x	x	x	x	x	x	x	x	x	x	x	x	x	
3	1031.5	MC [Asp^3^, Mdha^7^]MC-YR	x	x	x	x	x	x	x	x	x	x	x	x	x	x	x	x	x	x	
4	1024.6	MC [Asp^3^, Mdha^7^]MC-RR	x	x	x	x	x	x	x	x	x	x	x	x	x	x	x	x	x	x	
5	1010.6	MC [Asp^3^, Dha^7^]MC-RR	x	x	x	x	x	x	x	x	x	x	x	x	x	x	x	x	x		
6	981.5	MC [Asp^3^, Dha^7^]MC-LR	x	x	x	x	x	x	x	x	x	x	x	x	x	x	x	x	x		
7	973.5	CYP [Arg-Aph-Leu-MeTyr-Val-O-Thr]-Asp-HA																	x		
8	916.4	AP 915 [Ile-MeHty-HTyr-Val-Lys]CO-Tyr	x										x	x	x	x	x		x		x
9	910.5	AP G [Tyr-MeLeu-HTyr-Ile-Lys]CO-Arg	x	x	x	x	x	x	x	x	x	x	x	x	x	x	x	x	x		
10	858.5	OSC [Phe-MeAla-HTyr-Ile-Lys]CO-Tyr	x	x	x	x	x	x	x	x	x	x	x	x	x	x	x	x	x		
11	851.5	AP F [Phe-MeAla-HTyr-Ile-Lys]CO-Arg	x	x	x	x	x	x	x	x	x	x	x	x	x	x	x	x	x		
12	844.4	AP A [Phe-MeAla-HTyr-Val-Lys]CO-Tyr	x	x	x	x	x	x	x	x	x	x	x	x	x	x	x	x	x		
13	837.5	AP B [Phe-MeAla-HTyr-Val-Lys]CO-Arg	x	x	x	x	x	x	x	x	x	x	x	x	x	x	x	x	x	x	
14	828.4	AP D [Phe-MeAla-HTyr-Val-Lys]CO-Phe																	x		
15	801.4	PLC [Pro-Gly-Leu-Val-Met-Phe-Gly-Val]	x	x	x	x	x	x	x	x	x	x	x	x	x	x	x	x	x	x	
16	765.4	AER Cl-Hpla-Leu-(Xyl)Choi-Aeap						x	x				x	x	x	x	x	x			
17	749.4	AER Cl-Pla-Leu-(Xyl)Choi-Aeap												x	x	x					
18	731.4	AER Hpla-Leu-(Xyl)Choi-Aeap											x	x	x	x		x	x		
19	725.4	AER Cl-Pla-Leu-(Xyl)Choi-Agm																	x		
20	719.2	AER ?-Leu(Xyl)Choi-Agm											x				x	x	x	x	x
21	715.4	AER Pla-Leu-(Xyl)Choi-Aeap	x	x	x	x	x	x	x	x	x	x	x	x	x	x	x	x	x		
22	707.4	AER Hpla-Leu-(Xyl)Choi-Agm												x		x		x	x		
23	691.4	AER Pla-Leu-(Xyl)Choi-Agm					x		x	x			x	x	x	x		x	x		
24	643.3	AER Cl_2_-Hpla-Leu-Choi-Agm											x	x		x	x		x		
25	637.3	AER Cl-Hpla-Leu-Choi-Argal							x				x	x		x			x	x	x
26	625.4	Unknown			x		x	x	x	x			x	x		x		x	x		
27	621.3	AER ?-Leu-Choi-Agm	x		x			x					x	x		x	x	x	x		x
28	617.3	AER Pla-Phe-Choi-Aeap														x		x	x		x
29	609.3	AER Cl-Hpla-Leu-Choi-Agm		x	x			x					x	x		x	x	x	x		
30	587.3	AER ?-Leu-Choi-Agm	x		x		x	x	x	x	x	x	x	x	x	x		x			
31	583.3	AER Pla-Leu-Choi-Aeap												x	x	x					
32	561.4	AERMD (Pren)_2_Ile-Val-Pro-methiazole	x						x					x	x		x	x			
33	519.3	AER ?-Leu-Choi-Agm		x	x		x							x		x					

*AER* aeruginosin, *AERMD* aeruginosamide, *AP* anabaenopeptin, *CYP* cyanopeptolin, *OSC* oscillamide, *MC* microcystin, *PP* planktopeptin, *PLC* planktocyclin, ?—unknown residue

During the sampling campaign in 2009–2012, the total concentration of microcystins (MCs) in the reservoir always exceeded 4 µg/l and reached the highest values (22–23 µg/l) in September, when the biomass of *P. agardhii* was also the highest (Table 1). In all samples from the SDR, five microcystins were detected with LC–MS/MS system. On the basis of the *m/z* values and the fragmentation spectra of the pseudomolecular ion, the structures of the compounds were characterized as demethylated, Asp^3^-containing MC variants: [Asp^3^, Dha^7^]MC-RR (*m/z* 1,010), [Asp^3^, Mdha^7^]MC-RR (*m/z* 1,024), [Asp^3^, Mdha^7^]MC-LR (*m/z* 981), [Asp^3^, Mdha^7^]MC-RY (*m/z* 1,031) and [Asp^3^, Mdha^7^]MC-HtyR (*m/z* 1,045).

In other *Planktothrix*-dominated European lakes, the presence of at least one of the five demethylated MCs was also recorded (Barco et al. 2004; Welker et al. 2004a; Kurmayer et al. 2005; Briand et al. 2005; Welker and Erhard 2007; Bauman and Jüttner 2008; Tooming-Klunderud et al. 2008; Rohrlack et al. 2009; Rounge et al. 2009). Four of the microcystins (*m/z* 981, 1,024, 1,031, 1,045) were identified in field samples and in the *P. agardhii* isolates from Viry-Chãtillon’s lake in France (Yépremian et al. 2007). The same MCs isoforms were repeatedly detected during 33-year studies in *P. agardhii*-dominated Lake Steinsfjorden in Norway (Rohrlack et al. 2008). All these results confirm that production of demethylated MCs variants is a characteristic feature of *Planktothrix,* regardless of its origin.

The results of our four-year studies in the SDR showed a positive correlation between the total concentration of MCs and the biomass of *P. agardhii* (*r* = 0.74, *n* = 17, *p* = 0.003). Such a correlation was frequently reported for *Planktothrix* and *Microcystis* bloom samples (Briand et al. 2005; Izydorczyk et al. 2008; Monchamp et al. 2014). On the other hand, despite different environmental conditions that prevailed in the sampling seasons, the ratio of the total MCs concentration to *P. agardhii* biomass varied less than threefold (0.24 × 10^−3^ − 0.69 × 10^−3^, average 0.38 × 10^−3^ ± 0.15 × 10^−3^) (Table 1). Similar changes in MC production, but expressed as cellular quotas, were recorded by Briand et al. (2005) in *P. rubescens* from Luc du Bourget (France) (0.1–0.3 pg/cell). These changes were comparable to the changes observed in a single strains of cyanobacteria grown under different culture conditions (Sivonen and Jones 1999). Higher differences in MC cellular quotas were reported, e.g., for *P. agardhii* from Base Nautique de Viry in France (2–19 fg MC/cell) (Briand et al. 2008) or for *Microcystis* from Lake George in Uganda (0.03–1.24 fg MC/cell) (Okello et al. 2010). In the natural environment, the amount of MCs produced by the biomass of cyanobacteria depends on the proportion of MC producers in the cyanobacterial community. To some extent, it can also be modified by environmental factors such as light intensity, nutrient concentration, water pH, abundance of cyanobacteria and the presence of predators or viruses (Halstvedt et al. 2008; Briand et al. 2008; Tao et al. 2012, Agha et al. 2013). In the present work, the average production of MC by *P. agardhii* biomass (MC/Biomass, Table 1) in 2009 and 2010 was lower and statistically different than in 2011 and 2012 (one-way ANOVA, multiple comparisons test). On the other hand, in 2009 and 2010, the average biomass of *P. agardhii* was higher, compared to 2011 and 2012, but no statistically significant correlation with the MC/Biomass ratio was shown. Briand et al. (2008) and Sabart et al. (2010) reported higher MC cellular quotas at lower number of MC-producing cyanobacteria genotypes. On the basis of this finding, the authors suggested that microcystins may have some role in the development of the microorganisms under conditions suboptimal for their growth.

Apart from microcystins, in the cyanobacterial material, anabaenopeptins were frequently identified. Five of the seven detected anabaenopeptins (AP) were present in all bloom samples collected during our studies. The structures of the compounds were elucidated as AP A (Fig. 1) with molecular ion at *m/z* 844, AP B (*m/z* 837), AP E/F (*m/z* 851), oscillamide Y (*m/z* 858) and AP G (*m/z* 909). Of these peptides, AP B always showed the largest peak area in the chromatogram. AP 915 was less frequently encountered, and It was present in 41 % of the samples analyzed in years 2009–2012 (Table 2). With the respect to the produced anabaenopeptins, the *P. agardhii* population from the SDR was similar to *Planktothrix* populations from other European lakes. AP A, AP B, AP E/F and oscillamide Y were found, among others, in *Planktothrix* from Alpine lakes in Austria (Welker and Erhard 2007), Lake Steinsfjorden in Norway (Rohrlack et al. 2008), Lake Hallwilersee in Switzerland (Bauman and Jüttner 2008) and Lake Maxsee in Germany (Welker et al. 2004a). Similarly to the SDR, AP B together with [Asp^3^, Mdha^7^]MC-RR were present nearly in all samples from Fennoscandia lakes (Rohrlack et al. 2008, 2009).

In one of the anabaenopeptins that was detected only in the scums from the SDR, the amino acid Phe was located in the exocyclic position. The structure of the peptide was elucidated as AP D. Christiansen et al. (2011) analyzed the adenylation domain of the module in the anabaenopeptin gene cluster, which is responsible for the incorporation of the exocyclic amino acid into the AP structure. The authors found that a single strain of *Planktothrix* can coproduce APs with either Arg or Tyr in the exocyclic position 1. So far, AP D with Phe in this position has not been found in *Planktothrix*, but it is quite common in *Dolichospermum* (*Anabaena*) (Rouhiainen et al. 2010) and the brackish water *Nodularia spumigena* (Mazur-Marzec et al. 2013), which are characterized by a different organization of anabaenopeptin gene cluster (Rouhiainen et al. 2010; Christiansen et al. 2011). Therefore, we presume that the small amounts of AP D detected in scums in October 2012 may be attributed to the increased concentration of the peptide and/or the higher contribution of *Dolichospermum* or other AP D-producing cyanobacterial taxa in the sample.

Aeruginosins belong to the third group of peptides detected in the SDR. These compounds have a linear structure and are characterized by the presence of hydroxy-phenyl lactic acid (Hpla) or its derivatives at the *N*-terminus, the unique Choi residue (2-carboxy-6-hydroxyoctahydroindole) in position 3, and Arg or its mimetics at the *C*-terminal position (Fig. 2). *Planktothrix* can produce aeruginosins with chloride (*Cl*) and/or sulfate (*Su*) group located at Pla or Choi (Murakami et al. 1995; Welker et al. 2006; Welker and Erhard 2007; Cadel-Six et al. 2008). In the cyanobacterium, glycosylated aeruginosins (aeruginosides) with pentose sugar (xylose) (Fig. 2) were also found; however, the presence of the structures was rarely reported (Shin et al. 1997; Ishida et al. 2007, Welker and Erhard 2007). Aeruginosamide with *m/z* 561 was another peptide that occurred in a couple of samples from the SDR, but it was previously reported only in *Microcystis* (Lawton et al. 1999; Welker et al. 2004b). The structure of the peptide was elucidated as (Pren)_2_-Ile-Val-Pro-methiazole, where (Pren)_2_ stands for diisopronylamine (Fig. 1S).

In all cyanobacterial samples, the peptide with *m/z* at 801 was found (Table 2, Fig. 2S). The mass fragmentation spectrum of the compound corresponded to the MS/MS spectrum of planctocyclin (cyclo[Pro-Gly-Leu-Val-Met-Phe-Gly-Val]). This cyclooctapeptide was detected in bloom samples of *P. rubescens* from Lake Hallwilersee in Switzerland (Bauman and Jüttner 2008) and Lake Bled in Slovenia (Sedmak et al. 2008a). In the scums collected from the SDR during *P. agardhii* bloom, when a higher biomass of the material was analyzed, some other oligopeptides were detected (aeruginosin *m/z* 725, cyanopeptolines *m/z* 973 and planktopeptin *m/z* 1,126) (Table 2).

All samples from the SDR analyzed in this work were characterized by the presence of the same 12 peptides whose intensities in the LC–MS/MS chromatogram were the highest. With respect to these 12 compounds, the peptide profile in SDR was stable throughout the whole study period. However, the two isolated strains showed different peptide patterns (Table 2). In *P. agardhii* CCNP1325, seven oligopeptides were detected. These include 5 compounds that were present in all collected environmental samples: 3 microcystins, anabaenopeptin B and planktocyclin (Table 2). In contrast, *P. agardhii* CCNP1326 was “nontoxic” and produced five peptides (4 aeruginosins and anabaenopeptin 914), which were less frequently found in the reservoir. The differences in the peptide profiles of the two isolated strains strongly indicated that the population of *P. agardhii* in the SDR was not clonal and was composed of several chemotypes.

In the studies on *Planktothrix* populations from 23 European lakes, the three gene regions that are involved in the biosynthesis of microcystins (*mcy*B), aeruginosins (*aer*B) and anabaenopeptins (*apn*C) were analyzed (Kurmayer et al. 2011). The average proportions of the genes in green-pigmented *Planktothrix* populations were 31 ± 4 % (*mcy*B), 16 ± 2 % (*aer*B) and 43 ± 5 % (*apn*B). In our studies, the individual aeruginosin variants were detected less frequently than MCs and APs. This observation seems to support the above data indicating lower proportion of *aer*B in European *Planktothrix* populations, compared to *mcy*B and *apn*B (Kurmayer et al. 2011). On the other hand, aeruginosins were usually present in SDR at low concentrations. Therefore, it cannot be excluded that the decrease in the proportion of *aer*B genotype and/or the down-regulation of the *aer* genes led to lower concentrations of the peptide, which could not be detected with the applied method (detection limit 1.5 ng/ml). As a consequence, the peptide patterns determined by LC–MS/MS included only compounds which under existing environmental conditions were present in concentrations exceeding a defined limit of detection. Agha et al. (2013) used MALDI-TOF MS system in the analyses of oligopeptide patterns of three *Microcystis aeruginosa* strains grown under different culture conditions. Physiological stress affected the stability of oligopeptide profile among the strains in different ways and caused a gradual disappearance of microcystins with low intensity signals. The authors suggested that in view of these findings, the results of studies on chemotype diversity or stability of cyanobacterial populations should be interpreted with caution.

Besides the importance of the peptides as taxonomic markers, these compounds deserve attention due to their biological activity and potential ecological significance. Among the peptides, there are many inhibitors of key metabolic enzymes. Some aeruginosins and planktopeptins exhibit inhibitory activity against trypsin-type serine proteases even at nM level (Ishida et al. 1999; Grach-Pogrebinsky et al. 2003; Ersmark et al. 2008). Anabaenopeptins are also active toward proteolytic enzymes such as chymotrypsin, elastase and carboxypeptidase-A (Sano and Kaya 1995; Murakami et al. 2000; Itou et al.^1999^). Due to this kind of activities, the peptides may play some role in the interaction with other aquatic organisms, e.g., as elements of chemical defense against grazers or infecting agents (Rohrlack et al. 2003; Bauman and Jüttner 2008; Sedmak et al. 2008b; Sønstebø and Rohrlack 2011).

## Conclusions

During the four-year study (2009–2012), *P. agardhii* was the dominating species in the SDR. Microcystin concentration in the reservoir positively correlated with the biomass of the *P. agardhii*, while the changes in MC/Biomass ratio that were observed between the years were less than threefold. Production of microcystins, aeruginosins and anabaenopeptins, or even specific variants of the peptides, is common among the European *P. agardhii* populations, including those from Polish water bodies. The frequency of aeruginosins appeared to be lower than microcystins and anabaenopeptins. In all samples from the SDR, we detected the same 12 peptides, characterized by the highest signal intensity in LC–MS/MC chromatograms. However, the analyses of the two isolated strains indicated that *P. agardhii* population from the SDR was composed of several chemotypes characterized by different peptide patterns.

## Electronic supplementary material

Below is the link to the electronic supplementary material.

Supplementary material 1 (DOCX 34 kb)
