# Supplementary material for: Non-ribosomal peptides produced by Planktothrix agardhii from Siemianówka Dam Reservoir SDR (northeast Poland)
Source: Arch Microbiol. 2014 Jun 28;196(10):697–707. doi: 10.1007/s00203-014-1008-9 (PMC4168019; doi:10.1007/s00203-014-1008-9)
Supplement: Supplementary file 1 — Supplementary material 1 (DOCX 34 kb) [file 203_2014_1008_MOESM1_ESM.docx]

**Non-ribosomal peptides produced by *Planktothrix agardhii* from Siemianówka Dam Reservoir SDR (north-east Poland)**

Magdalena Grabowska, Justyna Kobos, Anna Toruńska-Sitarz, Hanna Mazur-Marzec

M. Grabowska, Department of Hydrobiology, University of Białystok, Świerkowa 20B

15-950 Białystok, Poland

J. Kobos, A. Toruńska-Sitarz, H. Mazur-Marzec (
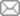
)

Institute of Oceanography, University of Gdańsk, Al. Marszałka Piłsudskiego 46

81-378 Gdynia, Poland

e-mail: [biohm@ug.edu.pl](mailto:biohm@ug.edu.pl)

tel.: +48585236621

fax.: +48585236712

Fig. S1 Mas fragmentation spectrum of aeruginosamide *m/z* 561 ((Pren)_2_Ile-Val-Pro-methiazole) produced by *Planktothrix agardhii* from the Siemianówka Dam Reservoir

Fig. S2 Mas fragmentation spectrum of planktocyclin *m/z* 801 ([Pro-Gly-Leu-Val-Met-Phe-Gly-Val]) produced by *Planktothrix agardhii* from the Siemianówka Dam Reservoir
